# Systematic identification of lincRNA-derived immunogenic peptides in melanoma

**DOI:** 10.1080/2162402X.2025.2538684

**Published:** 2025-08-02

**Authors:** Emilie Dupré, Amélie Guiho, Tiffany Beauvais, Léna Labous, Tristan Cardon, Corine Bertolotto, Amir Khammari, Gaelle Quéreux, Michel Salzet, Nathalie Labarrière, Catherine Rabu, François Lang

**Affiliations:** a INCIT, INSERM 1302, Labex IGO, Universitéd’Angers, Nantes Université, Nantes, France; b U1192 - Protéomique Réponse Inflammatoire Spectrométrie de Masse - PRISM, Univ. Lille, Inserm, CHU Lille, Lille, France; c Centre Méditerranéen de Médecine Moléculaire (C3M), INSERM U1065, Université Côte d’Azur, Nice, France; d Department of Dermato-Cancerology, CHU Nantes, Nantes, France

**Keywords:** Cancer immunotherapy, T cell epitopes, long intergenic non-coding RNA, melanoma, tumor antigens

## Abstract

The search for reliable shared tumor-specific antigens (TSAs) to improve cancer immunotherapy is on-going. The so-called non-coding regions of the genome have recently been shown to give rise to immunogenic peptides, including the melanoma-specific antigen MELOE-1 which is translated from the long intergenic non-coding RNA (lincRNA) *meloe* in an IRES-dependent manner. Here, we present a strategy to systematically identify tumor-specific antigens produced by ORFs within lincRNAs with IRES-like upstream structures. We provide evidence suggesting that in the melanocytic lineage a significant proportion of the selected lincRNAs can produce immunogenic peptides. T cell repertoires against some of these peptides were found in peripheral blood mononuclear cells (PBMCs) from healthy donors and melanoma patients, and in tumor-infiltrating lymphocytes (TILs) from metastatic melanoma patients. Finally, CD8+ T cell lines from melanoma patients specific for three of the characterized HLA-A *0201 epitopes could recognize melanoma cell lines, which were enhanced by reticular stress. Thus, these peptides may represent a new class of shared TSAs in melanoma and are attractive candidates for evaluation as targets for immunotherapy in preclinical studies. In addition, our selection strategy has the potential to identify new lincRNA-derived antigens in other cancers.

## Introduction

During the last 15 years, renewed interest in cancer immunotherapy has been fueled by encouraging clinical results with immune checkpoint inhibitors (ICI). However, response rates to ICI are suboptimal in most cancer types^1^ with a clinical response to PD1 blockade of approximately 35% to 40%. In this context, therapeutic vaccines that generate antigen-specific T cells appear to be attractive candidates for combination with ICI therapies.

To this end, efforts have been focused on tumor-specific antigens (TSAs). The dominant paradigm is that most TSAs originate from point-mutated exomic sequences (mTSAs) generating T cell neoepitopes that can be identified by whole exome sequencing of the tumor and MHC binding predictions.^2^ This is supported by observations that: (i) clinical responses to ICI correlate with tumor mutational burden and neoepitope recognition by T cells^3,4^ (ii) some neoepitopes were eluted from melanoma tissues^5^ (iii) vaccination with neoantigens in an adjuvant setting prevented relapse of melanoma^6,7^ (iv) adoptive cell transfer of lymphocytes directed against a neoantigen (mutated KRAS) induced partial tumor regression.^8^ However, the main drawback of these mTSAs is that they mostly result from passenger mutations,^9^ which are patient-specific and variably expressed in time and tumor location.^10^ In addition, despite the very high rate of mutations in melanoma, few immunogenic mTSAs have been identified by mass spectrometry analyses.^5^ This indicates that the absence of productive antigen processing constitutes a major limiting factor in the presentation of most of the predicted mutated neoepitopes.

Recent evidence suggests that other types of TSAs involved in tumor immunosurveillance may have been overlooked and hence the term “neoantigen” should be applied to a wider variety of TSAs. A report by Laumont *et al*. showed that a high proportion of TSAs derives from aberrantly expressed “non-coding” regions (aeTSA)^11^ representing approximately 75% of the genome.^12^ For example, indel mutations in microsatellite-unstable tumors can generate tumor-specific frameshifts that encode neoantigens with multiple T cell neoepitope.^13,14^ Similarly, immunogenic neoantigens may be produced by aberrant splicing events.^15,16^ In addition, recent reports suggest that long non-coding RNA (lincRNA)-derived peptides can be immunogenic^17^ and generate anti-tumor responses in mouse.^18^ However, to date, the presence of T cell repertoires directed against lincRNA-derived peptides in patients with cancer has not been reported.

We previously identified the polypeptide MELOE-1 as an immunogenic antigen in metastatic melanoma patients^19^ and found that it was translated in a tumor-restricted, IRES-dependent manner from a short ORF of the lincRNA *meloe*.^20^ We documented the presence of a vast public T-cell repertoire against the immunodominant peptide of this antigen in patients with metastatic melanoma.^21,22^ Finally, we showed that, in contrast to the high immunogenicity of MELOE-1, another peptide, coined MELOE-3, a product of the cap-dependent translation of a proximal ORF from lincRNA *meloe*, was expressed in both melanocytes and melanoma cells and was poorly immunogenic.^23^ This prompted us to exclude peptides that might be expressed through cap-dependent translation.

Here, we present a strategy to identify lincRNAs within the melanocyte lineage that produce immunogenic peptides through unclassical translation.

## Material and methods

### Cell culture

Tumor cell lines, the human TAP-deficient cell line T2 and the HLA-A *0201 B-EBV cell line were cultured in RPMI 1640 medium with 100 U/mL penicillin, 0.1 mg/mL streptomycin, 2 mM L-glutamine (standard medium) (Gibco, France) and 10% fetal bovine serum (FBS) (Eurobio). Melanocytes were cultured in Medium 254 (Gibco) supplemented with 2 mM L-glutamine and 1% human melanocyte growth serum. T cells were cultured in standard medium with 8% human serum (Panbiotech) and IL-2 (150 UI) (Proleukin, Novartis). Tumor Infiltrating Lymphocytes (TILs) were obtained from the Gene and Cell Therapy Unit (UTCG, Nantes, France) from invaded lymph node of metastatic HLA-A *0201 melanoma patients (biocollection from Nantes Hospital DC-2008–402).

### Total RNA extraction

RNA was extracted from three melanocyte cell-lines and three melanoma cell-lines (M383 WT, M305BRAF V600E and M315 NRAS p.Q61R) at low passages for deep sequencing, and from various cancer cell-lines to perform quantitative PCR on selected transcripts. Briefly, cells (3-5x10^5^ were homogenized in RLT buffer and total RNA (biological duplicates for each line) was extracted according to the manufacturer’s protocol (RNeasy micro kit, Qiagen). DNA was removed with an RNase-Free DNase Set (Qiagen), and their purity and concentration were assessed using a Nanodrop-1000 spectrophotometer (ThermoFisher Scientific).

### RNA sequencing

RNA sequencing was performed by Helixio (Hybrigenics, France). cDNA libraries were made from 400 ng of quality-controlled RNAs (RIN values > 7.0) using NEBNext kits according to the manufacturer’s instructions (New England Biolabs). Sequencing was performed using the NextSeq500 system (Illumina), with 75bp single-read at a depth of 40 million reads. The quality control, quantification and identification are detailed in the supplementary file. The entire transcriptome contained 184,588 identified transcripts and 15,118 unknown transcripts.

### In silico selection

#### lincRnas selection

Only transcripts with FPKM values ≥ 0.1 in at least 4/6 samples, identified as lincRNAs in the Ensembl database (1309 candidates) and longer than 1000nt (440 candidates) were selected. The FPKM threshold was set according to the level of expression of the lincRNA *meloe* used as an internal control (FPKM = 0.132). Their expression levels were compared with the expression levels in four tumor types reported in the TCGA data bank: breast carcinoma (*n* = 1222), colon carcinoma (*n* = 514), ovarian adenocarcinoma (*n* = 379) and mesothelioma (*n* = 86). Fold change (FC) was calculated for pre-selected lincRNAs in melanoma ((FPKM melanocytic lineage-FPKM cancer type)/FPKM cancer type), and only those that had FC > 2 were retained (59 candidates). The use of public databanks is approved by Nantes university ethical committee.

#### ORF and epitope prediction

ORFs shorter than 105nt and the first ORFs over 105nt were excluded. Secondary structures within the 200 nt sequence upstream of each of the 153 selected ORFs (from 53 lincRNAs) were modelized using the online mfold algorithm (http://www.unafold.org). Putative IRES sequences were selected based on ΔG values between −90 and −30 and the presence of stem-loop structures (63 ORFs were selected). The presence of putative HA-A *0201 epitopes was predicted using NetMHCpan 4.1 server.^24^ We designed 51 long peptides that encompassed the predicted epitopes (Table S1).

### RT-qPCR

Total RNA was extracted from different cancer cell lines. DNAse-treated RNA (1 µg) was reverse-transcribed using an oligo dT primer (RevertAid H Minus Reverse Transcriptase). 25ng of cDNA was used as a template for qPCR analysis (Master Mix SYBR PCR) on an M×3000 apparatus (Stratagene). The mean threshold cycle (Ct) values from triplicate qPCR were normalized to the mean Ct value of RPLP0. The relative expression of transcripts for each cell line was further normalized to the mean expression of all positive melanoma samples (2^−∆∆Ct^ method).

### Peripheral blood mononuclear cells (PBMCs), monocytes isolation and DC

Whole blood was obtained from HLA-A *0201 healthy donors (Etablissement Français du Sang, Nantes, France) or from HLA-A *0201 metastatic melanoma patients before the initiation of anti-PD1 therapy, patients having signed written informed consent approved by Nantes Hospital ethical committee (number DC-2011–1399). PBMC were isolated using density gradient centrifugation. Monocytes were purified using counterflow centrifugal elutriation and purity was checked by CD14 mAb staining (clone TÜK4, Myltenyi Biotech). Immature dendritic cells (iDC) were generated by culturing monocytes in RPMI supplemented with 2% albumin (Vialebex), 1000 UI/mL GM-CSF and 200 UI/mL IL-4 for 5 days. iDC were then matured with 20 ng/mL TNFα and 50ug/mL Poly I.C. Mature DC were CD80+, CD86+, HLA-DR + (stained with clones 2D10, IT2.2 and L243, respectively, Biolegend).

### In vitro stimulation and expansion of T cells

PBMC were plated in 96 round bottom wells (2 ×10^5^ cells/well) in RPMI supplemented with 8% human serum and 50 UI/mL IL-2. A pool of 2–3 candidate synthetic long peptides (SLP) at 5 µM (85% pure) (GeneCust) was added to each well. Cultures were supplemented on day 1 with 1000 U/mL GM-CSF and 500 UI/mL IL-4 for DC differentiation and on day 2 with 1000 UI/mL TNFα, 10 ng/mL IL-β1 and 1 µM prostaglandin E2 for DC maturation according to the published accelerated DC protocol (acDC).^25^

### Microcultures screening, characterization of response and SLP identification

At day 21, autologous DC unpulsed or pulsed with a pool of three SLP were added to each microculture in the presence of brefeldin-A (10 µg/mL) for 5 h. Microcultures containing specific T cells were detected by intracellular staining with anti IFN-γ mAb (clone B27) and CD8 surface staining (clone RPA-T8) (BioLegend). Positive microcultures were rechallenged with an HLA-A *0201 B cell line loaded with each individual SLP from the initial pool (5 µM) to identify the stimulating SLP. HLA A * 02 restriction was confirmed by blocking recognition with HLA-A2 specific mAb BB7.2 at 40 µg/ml. HLA-A *0201-restricted epitope was confirmed using peptide-loaded (5 µM) TAP-deficient HLA-A *0201 T2 cell line. Flow cytometry analysis was performed using a Canto I-HTS (BD Biosciences).

### Expression of linc-derived T cell epitopes by melanoma cell lines.

To evaluate the expression of the HLA-A *0201-restricted epitopes VS17p, SF15-dec1 or SF15-dec2 at the surface of melanoma cells, T-cell populations specific for each epitope were co-cultured for 5 h in the presence of brefeldin A (10 μg/mL, Sigma) with a panel of HLA-A *0201 positive melanoma cell lines or the HLA-A *0201 negative M6 cell line as control at an E/T ratio of 1:2. T cells were then stained with a PE-conjugated anti-CD8 mAb (BioLegend), and intracellular-stained with an APC-conjugated anti-TNFα mAb (clone Mab11) before flow cytometry analysis. To explore the effect of stress, melanoma cell lines were pretreated or no with thapsigargin (200 nM, Sigma-Aldrich) for 24 h and washed extensively over another 24 h period before co-culture with the T cell lines.

### Mass spectrometry

Mass spectrometry was used to ascertain the translation within melanoma cells of the selected ORFs. The proteins corresponding to these ORFs were digested in silico to produce specific target peptides. Spectra acquired from the synthetic target peptides allowed us to determine the retention time and specific ion transitions for each targeted protein. Identification of these transitions, correlated with the retention time, within tumor lysates was performed to confirm the translation of these peptides in tumor cells. NanoLC MS/MS analysis was performed using a Thermo Easy-nanoLC-1200 system followed by analysis on an Orbitrap Eclipse mass spectrometer with a nano-electrospray ionization (nESI) source. The settings used for the analysis are described in supplemental data files.

### ELISPOT assay

An IFN-γ ELISPOT assay was performed on enriched CD8^+^ TILs obtained from melanoma-invaded lymph nodes of HLA-A *0201 patients (originating from our registered biocollection PC-U892-NL).

In brief, 2.5 10^5^ enriched CD8^+^ TILs were seeded in ELISPOT 96 wells (Mabtech) in triplicate with HLA-A *0201 epitopes (Table 1) and incubated for 24 h at 37°C and 5% CO_2_. After washing, the plates were read according to the manufacturer’s instructions. IFNγ spots were counted using an ELISPOT reader (Bio-Sys’Bioreader), and the number of spot forming units (SFU)/10^6^ T cells was calculated from the mean of triplicates after subtraction of the background (no peptide).

**Table 1.** Selected lincRnas and corresponding immunogenic synthetic long peptides (SLPs). SLP name, sequence, the ORF and the corresponding transcript are indicated. The presence of CD4 and/or CD8 T cell responses in *in vitro*-stimulated healthy donor PBMCs is indicated. The last column shows the minimal immunogenic HLA-A *0201 identified epitopes. For each ORF, the mean of the 3 first γG values of the 200bp upstream of the ATG as predicted by mfold is indicated.

lincRNA	Transcript	ORF	Putative IRES (γG)	SLP	SLP sequence	CD4 responses	CD8 responses	Confirmed HLA-A *0201 epitope(s)
AC024909	ENST00000611513	ORF 841–1272	−78.9	SS16	SSCSLSHATVLLIAIS	1/2	0/2	
LINC00518	ENST00000496285	ORF 549–905	−62.90	SF15	SPPCLWYFLPTLGCF	0/3	2/3	CLWYFLPTL/PCLWYFLPTL/CLWYFLPTLG
C11orf72	ENST00000333139	ORF 242–997	−61.93	VS17	VISHLKLTMYPWGLPPS	1/3	2/3	TMYPWGLPPS
				PH16	PQFQLLWLCPYKLDLH	2/3	1/3	LLWLCPYKL
		ORF 1191–1346	−43.03	MS16	MCFVKQMLEGSMLVKS	1/3	1/3	
AL671710	ENST00000610245	ORF 1087–1356	−34.83	VT17	VSPLLLISHEWHLVWAT	1/3	1/3	
LINC00589	ENST00000506121	ORF 926–1129	−66.27	GL16	GIRILSPPLWVLTHDL	1/3	2/3	
AL390066	ENST00000456414	ORF 528–875	−47.27	EE17	EGTMWMGVQLLDWGCTE	1/3	1/3	
LINC00839	ENST00000429940	ORF 1523–1753	−47.93	YT17	YCIFLPRLVSNVETAHT	0/2	1/3	
AL445686	ENST00000577528	ORF937–1260	−29.60	ME17	MGIEYMISKVTFTSVIE	2/3	1/3	
AC004951	ENST00000447643	ORF920–1465	−45.63	VE17	VCLGWLVSGGSFVCQAE	1/2	0/2	
				SG17	SETLFPQTFQKLVEHGG	1/2	0/2	
AC131254	ENST00000517356	ORF 245–370	−48.96	GT16	GPRWLSSQHTFAVTCT	1/2	0/2	
LINC00473	ENST00000584911	ORF 479–583	−58.23	DK19	DMRVSILWRGSFYFLSSTK	1/2	0/2	
AP001107.1	ENST00000501708	ORF 494–778	−63.57	LL16	LASPVLFRADVLVAGL	1/2	0/2	
AC084018.2	ENST00000613093	ORF 539–862	−84.97	KS17	KALFIFLTYPLSPVPWS	2/4	2/4	
AC121247.1	ENST00000440528	ORF 529–969	−45.33	LK17	LPAPLLSLLGFIACARK	1/2	1/2	
AC006946	ENST00000608373	ORF 499–690	−65.20	RK17	RAPTLKLAFLLFLRFVK	0/2	1/2	
AC136944.2	ENST00000568752	ORF 2483–2848	−50.23	GV17	GLAVHWPFLPWPCPSLV	0/3	1/3	FLPWPCPSLV

## Supplemental methods

### Supplemental data on RNA deep sequencing

RNA sequencing was performed by Helixio company (Hybrigenics, St Beauzire, France). Briefly, RNA quality was checked by Nanodrop ND-1000, fluorimeter Qubit 2.0 (Thermo Fisher Scientific) and Bioanalyzer 2100 (Agilent Technologies). cDNA libraries were made from 400 ng of total RNAs (RIN values > 7.0) with NEBNext kits according to manufacturer’s instructions (New England Biolabs). Sequencing was performed using NextSeq 500 system (Illumina), 75 bp single-read at a depth of 40 millions of reads. Quality of reads was confirmed with FastQC v0.11.3 software (Babraham Institute), with a Phred quality score > 34 for each sample. The total number of reads was comprised between 37.9 million to 51.0 million and %GC was between 48% and 51%. Reads were aligned to a reference genome (GRCh38, release 91) using Bowtie and TopHat. More than 95% of reads were aligned with the reference genome. Quantification was carried out using Ensembl annotation file and Cufflinks software, giving FPKM values (Fragments per Kilobase Per Million mapped reads). Transcriptome was reconstituted using Stringtie and expression values were normalized using Cuffdiff software (Cufflinks). The whole transcriptome experiment contained 184 588 identified transcripts and 15,118 unknown transcripts.

### Supplemental data on MS/MS analysis

#### Sample digestion

Filter-Aided Sample Preparation (FASP) of tumor cell lysates was conducted using a 30 kDa cutoff Amicon filter. Briefly, reduction was done adding 100 μL of 100 mM Dithiothreitol (DTT) in denaturing buffer (8 M Urea, 100 mM Tris-HCl, pH 8.5) at 56°C for 40 minutes. Samples were transferred to the Amicon filter, concentrated by centrifugation (14,000 g for 15 minutes). Alkylation was performed by addition of 100 μL of 50 mM Iodoacetamide in denaturing buffer at room temperature for 30 minutes in the dark. Subsequently, 40 μL of 40 ng/μL Trypsin, Mass Spec Grade, was added to the filter and incubated overnight at 37°C. The resulting peptides were then acidified with 0.1% TFA and vacuum dried.

#### NanoLC-MS/MS analysis

Samples were reconstituted in 10 µL of 0.1% TFA. Solid-phase extraction was performed using C-18 Ziptips (Millipore, Saint-Quentin-en-Yvelines, France), and peptides were eluted with ACN/0.1% TFA (8:2, v/v) and dried for storage. Before analysis, samples were resuspended in 20 µL of ACN/0.1% FA (2:98, v/v), and 10 µL were injected for analysis. Separation was carried out using a Thermo Easy-nanoLC-1200 system with an Acclaim PepMap trap column and an Acclaim PepMap RSLC C18 analytical column (Thermo Scientific). Peptides were separated at a flow rate of 300 nL/min. The LC gradient parameters were as follows: 6 to 11% B (ACN) to 0–20 minutes, increasing to 16% B at 30 minutes, 21% B at 40 minutes, 26% B at 50 minutes, and 37% B at 60 minutes, followed by a rise to 100% B at 65 minutes. An Orbitrap Eclipse mass spectrometer was used for MS acquisition with a nano-electrospray ionization (nESI) source. The spray voltage was set to 1.8 kV for positive ion mode at 275°C. The initial MS scan was performed at 60,000 resolution (FWHM), covering a scan range of 350–1600 m/z. The automatic gain control (AGC) target was set to 600,000 with a maximum injection time of 200 ms, and one microscan was acquired per spectrum. For MS/MS analysis the collision energy was fixed at 40% with HCD activation. The Orbitrap detector was used at a resolution of 60,000 covering a mass range of 150–1700 m/z. FAIMS voltages were applied at −40 V for SRM5. The Parallel Reaction Monitoring (PRM) method was configured to target the following transitions: SRM5THGPYVITGDYPR, which encompasses the precursor ions with m/z values of 738.3675, 738.869, 739.3703, 492.5808, 492.9151 and 493.2493 at a charge state of + 2 and + 3.

#### Peptide data analysis

The targeted proteins were digested in-silico to produce the specific target peptides and spectra acquired from synthetic peptides allowed us to determine the retention time and specific ion transitions for each targeted protein. The identification of these transitions, correlated with the retention time, confirms the presence of the peptide in a complex sample. This analysis was carried out using Skyline software (version 22.2).

## Results

### Identification of long non-coding RNAs in the melanocytic lineage

**Figure 1.**
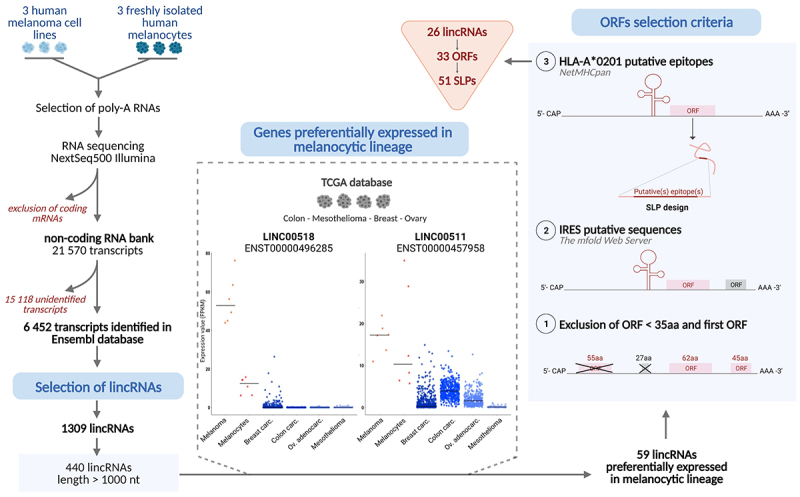
Pipeline of in silico selection of lincRNA, ORF and SLPs. After deep sequencing of poly-A RNA of 3 melanoma and 3 melanocytes cell lines, coding transcripts were excluded and lincRnas > 1,000nt were selected. 59 candidates were further selected for their high expression. ORF selection: i) exclusion of the first ORF and of ORFs < 35 aa, ii) presence of a putative IRES structure within the first 200 nt upstream of the ATG. Within these 33 ORFs, 51 putative HLA A * 0201 epitopes were identified and used to design SLPs.

To identify long non-coding RNAs (lincRNAs) that are shared by melanoma cell lines and normal melanocytes, we performed RNA deep sequencing on poly-A RNAs from three freshly isolated human melanocytes and three melanoma cell lines recently established from metastases of melanoma patients. Regarding the Raf pathway, one cell line was not mutated, one had Braf V600E mutation and the third harbored an N-ras Q61R mutation. These different mutational statuses were selected to assess expression of lincRNAs independently of the presence of driver mutation.^26^ The “non-coding” RNA library contained 21,570 transcripts among which 6,452 were present in the Ensembl database. We focused on 1,309 transcripts identified as lincRNAs and selected those that were over 1,000 nt long to increase our chances of finding translated ORFs. The 440 lincRNAs were compared for expression in the melanocytic lineage (as determined by FPKM) versus other cancer types – colon cancer, mesothelioma, breast cancer and ovarian cancer as reported in The Cancer Genome Atlas database. We focused on lincRNAs that were significantly expressed in the melanocytic lineage (Figure 1) and selected 59 lincRNAs. Two typical gene expression profiles are presented in Figure 1 with LINC00518 was exclusively expressed in the melanocytic lineage, whereas LINC00511 was also expressed in other cancers.

### Design of 51 long synthetic peptides from selected ORFs

Among the 59 selected lincRNAs, we searched for ORFs that may be translated by non-canonical translation into peptides at least 35 aa in length (Figure 1) to increase the probability that the selected ORF contains HLA-A *0201 CD8 epitopes. We also systematically discarded the first ORFs over 105 nucleotide long to exclude ORFs that may be expressed through cap-dependent translation. The 53 selected lincRNAs contained 153 such ORFs. To identify putative IRES sequences, we performed secondary structure analyses on the 200 nucleotides upstream of these ORFs, using the on-line RNA mfold algorithm (http://www.unafold.org).^27^ For each ORF, we examined the three most likely RNA folds, since the structure with the best prediction is not always the most thermodynamically stable^28^ and averaged the ΔG of these three predictions. We selected 63 ORFs with upstream regions containing secondary stem-loop structures with ΔG between −84,9 and −29,6 (mean γG = −55.15) based on the comprehensive analyses of IRES reported by Weingarten-Gabbay et al.^29^ These 63 ORFs were translated *in silico* into peptides and we looked for potential HLA-A *0201 epitopes using the NetMHCpan 4.1 server.^30^ The 51 epitopes predicted as HLA-A2 binders (weak or strong) were derived from 33 ORFs from 26 lincRNAs (Figure 1).

Since this reverse immunology approach may select epitopes that are not actually processed,^31^ we designed long synthetic peptides (SLP) by extending the aa sequence upstream and downstream of the HLA-A *0201 predicted epitopes to make processing of these SLP a prerequisite for presentation by antigen-presenting cells (APC). Thus, we designed 51 SLPs ranging between 15 and 19aa, containing one or more HLA-A *0201 predicted epitopes (Figure 1 and table S1).

### 19 of the 51 SLPs from lincRNAs are immunogenic

To evaluate the immunogenicity of our SLPs, we studied their ability to recall specific T cell responses *in vitro* in healthy HLA-A *0201 donor PBMCs. Based on our previous observations that a MELOE-1-specific T cell repertoire is present in both healthy individuals and melanoma patients,^21^ our hypothesis was that these peptides from lincRNAs may be abnormally translated in melanocytes that were stressed and/or undergoing oncogenic transformation and thus generate specific memory T cells also in some healthy individuals.

**Figure 2.**
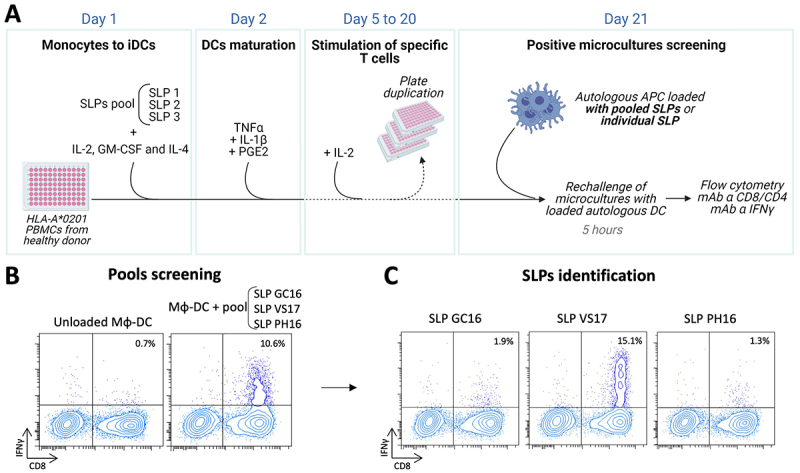
Amplification of specific T cells and SLP identification. A. Schematic representation of *in vitro* specific T cell expansion. HLA-A *0201 PBMCs were seeded in 96 –well plates with pools of 2 to 3 SLPs in the presence of GM-CSF, IL4 and IL2. On D2, a DC maturation cocktail was added. Fresh IL-2 (50UI/ml) was replaced as needed until screening at D21. Microcultures were restimulated for 5 h with Mφ-DC pulsed with pools of SLPs and IFNγ secretion was assessed. B. Example of a positive microculture reacting against a pool of three SLP identified by IFNγ intracellular staining. C. Within positive pools, individual immunogenic SLP were identified by restimulation for 5 h with a SLP-pulsed HLA-A *0201 B cell line.

PBMCs from 2 or 3 healthy HLA-A *0201 donors were cultured with pools of three SLPs in the presence of DC differentiation and maturation agents adapted from the previously published accelerated DC protocol^25^ (see M&M). At day 21, microcultures were restimulated with autologous APCs loaded with the SLP pools, and IFN-γ secretion was measured (Figure 2A). Pool screening identified microcultures containing CD8^+^ (Figure 2B, top) and/or CD8^−^ (Figure 2B, bottom) T cell responses. It should be pointed out that in these culture conditions, priming of naïve T cells did not occur but only revealed memory responses.^25^

Positive microcultures were then rechallenged with each single SLPs to identify which one was immunogenic (Figure 2C). Figure 2B,C show typical examples of immunogenic SLP within the pool.

Amongst the 51 SLPs tested, 13 SLPs generated IFN-γ positive HLA-A *0201-restricted CD8 T-cell responses in at least one healthy donor’s PBMC sample. Nine of them also stimulated CD4 T-cell responses, while 6 SLPs only triggered CD4 T-cell responses (Table 1). The observed CD4 responses were fortuitous since SLPs were selected for the presence of putative HLA-A *0201-restricted epitopes only; thus, HLA class II restriction of the CD4 responses was not investigated.

**Figure 3.**
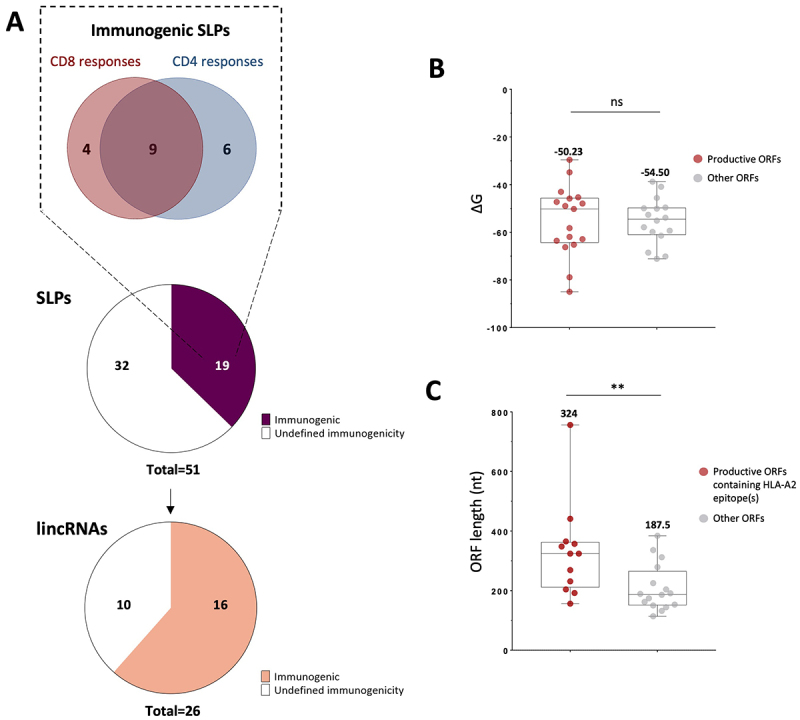
A high proportion of selected lincRnas code for immunogenic SLPs. A. 19 out of 51 SLPs tested were immunogenic, originating from 16 lincRnas. B. No difference in stability (γG) of the 200 nt secondary structures upstream of the selected ORFs between ORFs that produced CD8 and/or CD4 epitopes (red) and ORFs that did not (gray). C. ORFs producing HLA-A *0201 epitopes (red) are significantly longer than the non-producing ones (*p* < 0.01, Mann-Whitney test).

Altogether, 19 out of 51 SLPs (37.2%) were immunogenic and came from 16 out of the 26 lincRNAs (61.5%) analyzed (Figure 3A). For each of the 16 lincRNAs that produced immunogenic peptides, we verified that among the reported alternative transcripts in the Ensembl database, no alternative splicing would place the expressed ORFs at the 5’ end of the transcript and thus allowing cap-dependent translation (not shown). In addition, we found that transcript C11orf72 could translate two distinct ORFs to produce three immunogenic peptides (ORF_242–997_: VS17 and PH16 and ORF_1191–1346_: MS16). No significant correlation between γG of ORFs and the detected T cell responses was observed (Figure 3B). In contrast, we observed significant differences in length between the 12 ORFs for which HLA-A2-restricted responses were detected compared to ORFs with no response (median 324 nt vs 187.5 nt, *p* < 0.01) (Figure 3C).

### Identification of minimal SLP HLA-A *0201 epitopes

To explore the CD8^+^ T cell repertoires against these new antigens in patients, identification of the minimal HLA-A *0201 epitopes of the selected SLPs was required. We first verified HLA-A *02 restriction by blocking T cell recognition of the SLPs with the HLA-A *02 specific BB7.2 antibody (figure S1). We then loaded the predicted HLA-A *0201 epitopes onto the HLA-A *0201 tap-deficient T2 cell line and rechallenged the SLP reactive microcultures (figure S1).

Using this procedure, the nonamer LLWLCPYKL from SLP-PH16, decamer TMYPWGLPPS from SLP-VS17, and decamer FLPWPCPSL from SLP-GV17 were identified. We also identified three epitopes from SLP-SF15, nonamer CLWYFLPTL, decamer PCLWYFLPTL and decamer CLWYFLPTLG that stimulated distinct SLP positive microcultures from the same donor suggesting that the T cell repertoires against these three epitopes may be different. Thus, we retained all of them for subsequent analyses. Altogether, we characterized as yet 6 minimal HLA-A *0201 epitopes from 3 different lincRNAs (LINC00518, AC126944.2, C11orf72) recognized by CD8^+^ T cells from healthy donors (Table 1).

**Figure 4.**
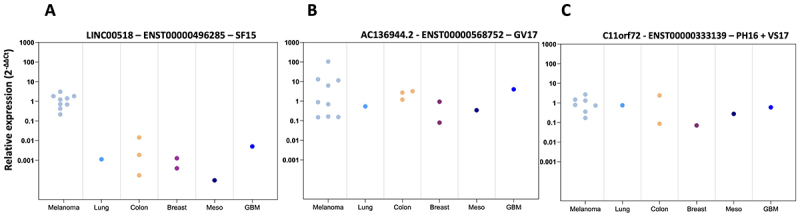
RT-qPCR of immunogenic transcripts in various cancer cell lines. Relative expressions of each transcript, normalized to RPLPO gene, (panel a for linc00518, panel B for transcript AC136944.2 and panel C for transcript C11orf72) were assessed in cancer cell-lines from various tissues (LUD, lung cancer; SW601, SW480, HCT116: colon; MCF7, MDA-MB-231: breast; Meso34/mesothelioma; U251 for glioblastoma (GBM)) compared to the mean expression within melanoma cell lines.

Before proceeding further, we checked the expression of each of the corresponding transcripts by quantitative PCR in various cancer cell lines. We chose primers that selectively amplified only the relevant transcripts amongst the alternative transcripts described for each of these 3 lincRNAs. We confirmed that the main transcript from LINC00518 was highly expressed in the 9 melanoma cell lines tested and weakly in all the other cancer cell lines (Figure 4A). In contrast, the transcripts from AC126944.2 and C11orf72 were expressed at comparable levels in all the cancer cell lines tested (Figure 4B,C).

### Melanoma patient-derived tumor-infiltrating lymphocytes are specific for lincRNA-derived antigens

To assess the relevance of these specific T cell responses against the six identified epitopes in melanoma patients, we performed ELISPOT assays on CD8^+^ tumor-infiltrating lymphocyte (TIL) populations from 22 HLA-A *0201 melanoma patients from a previous clinical trial.^32,33^

**Figure 5.**
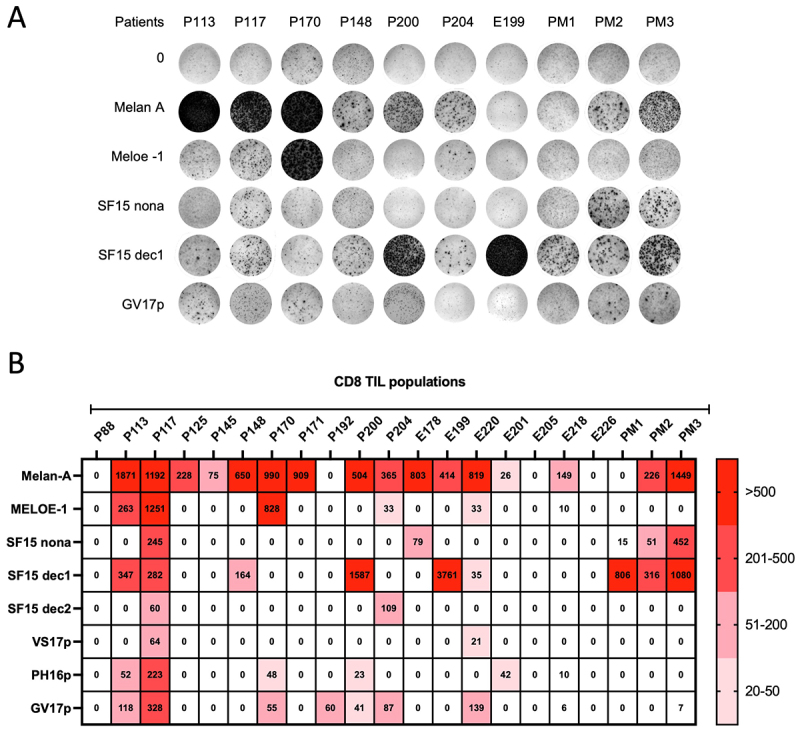
Detection of specific CD8^+^ T lymphocytes in TILs from HLA-A *0201 melanoma patients. A. Example of an IFNγ ELISpot assay performed on TILs stimulated with the indicated HLA A * 0201 epitopes. Unstimulated cells were used as negative controls. B. Heat map representing the number of spots/10^6^ TIL (above background) for each epitope in 22 patients.

We evaluated the production of IFNγ in response to the 6 minimal epitopes along with the HLA-A *0201 epitopes from MELOE-1 and Melan-A as positive controls. Typical examples are shown in Figure 5A and a summary of all the experiments is shown in Figure 5B. All six new epitopes were recognized by at least one TIL population. Together, the three SF15 epitopes activated TILs from 11 patients, with a strong predominance of responses toward SF15-dec1 compared to SF15-dec2 and SF15 nona. TIL responses toward epitopes from VS17 and PH16 from C11orf72 were less frequent, whereas the GV17 epitope generated reactivity in eight patients. Finally, TILs from patient 117 contained specific T cells against all 6 new epitopes in addition to MELOE-1 and Melan-A epitopes, and this patient was one of those who did not relapse following re-injection of TILs in the above-mentioned ACT trial.

Altogether, these data demonstrate the presence of T cells specific for lincRNA-derived epitopes in TILs from metastatic melanoma patients, suggesting that these neoantigens may participate in the T cell immune response against melanoma.

### T cell repertoires against lincRNA-derived antigens are present in the blood of melanoma patients

**Figure 6.**
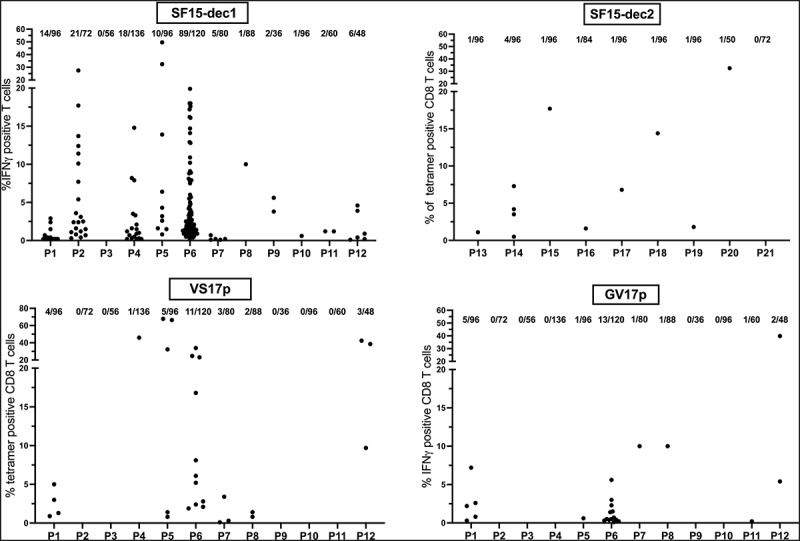
Detection of specific CD8^+^ T lymphocytes in PBMCs from HLA-A *0201 melanoma patients. PBMCs were stimulated with indicated HLA-A *0201 epitopes in 96 well plates and rechallenged at D21. Specific T cells were detected either by tetramer staining or by IFNγ production. The proportion of positive microcultures for each patient is indicated.

To buttress our hypothesis that these neoepitopes may be involved in the immune response against melanoma, we evaluated the frequency of specific T cell repertoires against the most promising epitopes, -SF15dec1, VS17p and GV17p- in the blood of melanoma patients. We collected PBMCs from 12 metastatic melanoma patients, stimulated them *in vitro* with the selected HLA-A *0201 epitopes and measured specific CD8 T-cell responses following restimulation either by IFNγ production (SF15-dec1 and GV17p) or tetramer staining (VS17p). An additional 9 patients (P13 to P21) were tested for reactivity against SF15-dec2. The frequency of microcultures containing reactive T cells and the percentages of positive cells in each well of each melanoma patient after *in vitro* peptide stimulation of their PBMCs (as previously described) are shown in Figure 6. A T cell repertoire directed against SF15-dec1 was found in the peripheral blood of 11/12 patients, with some patients exhibiting very high frequencies of reactive T cells. T cells reactive against SF15-dec2 were present in 8/9 other patients, but at lower frequencies than those with SF15-dec1. T cell repertoires against VS17p were also frequently found in PBMCs (7/12 patients), while VS17 reactivity was rare among TILs (as described above). Reactivity against GV17p was detected in PBMCs from 6/12 patients, similar to that found in TILs. Nonetheless, a comparison between the frequencies observed among PBMCs and TILs in metastatic melanoma patients should be made with caution since many immunological events may have occurred within the tumor environment that could explain these discrepancies.

### T lymphocyte cell lines specific for lincRNA-derived epitopes can recognize melanoma cell lines

We generated T lymphocyte populations specific for the HLA-A2 epitopes PCLWYFLPTL (SF15-dec1) or CLWYFLPTLG (SF15-dec2) from LINC00518, or TMYPWGLPPS (VS17p) from C11orf72. These cell lines were obtained by tetramer sorting (VS17p and SF15-dec2) after peptide stimulation of PBMCs from melanoma patients. In the case of SF15-dec1, tetramer synthesis was unsuccessful; thus, the corresponding T cell line could not be sorted, but was nevertheless more than 80% reactive. The TNFγ response curves of the three T cell lines toward epitope-loaded T2 cells are shown in Figure S2.

**Figure 7.**
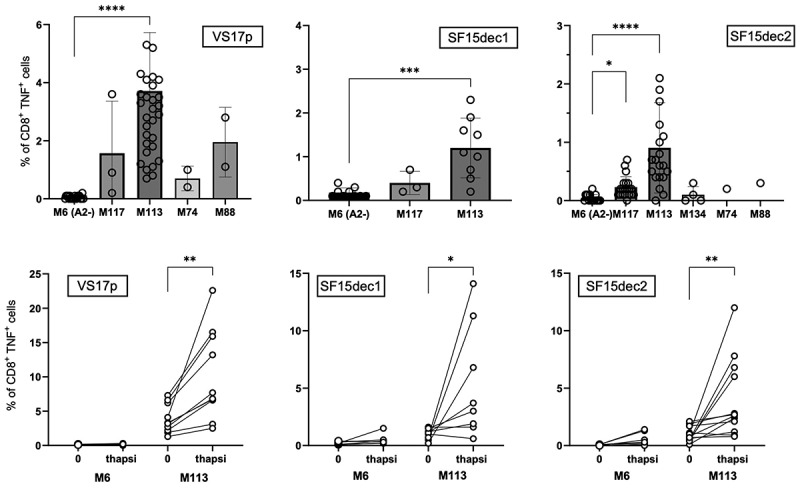
Expression of lincRNA-derived epitopes on melanoma cell lines. A. A panel of melanoma cell lines were co-cultured for 5 h with T cell lines specific for VS17p, SF15-dec1 or SF15-dec2 and melanoma recognition was assessed by intracellular TNFγ staining. The HLA-A *0201 negative melanoma cell line was used as control. ****p < 0.0001, Kruskal-Wallis test. B. M113 and M6 were treated with thapsigargin (200 nM) for 24 h before co-culture with the specific T cell populations and recognition was assessed as in A. **p < 0.01, Wilcoxon paired test. C. A representative experiment showing TNFγ production of the VS17p-specific T cell population after co-culture with melanoma cell-line M6 (negative control) or M113 melanoma cell-line, pre-treated or not with thapsigargin. Dot plots are shown after gating on CD8+ cells to exclude melanoma cells from the analysis.

We explored the ability of a panel of melanoma cell lines to spontaneously stimulate these specific cell lines. As shown in Figure 7A, we consistently observed a low but significant percentage of TNFγ-producing T cells when challenged with the different HLA-A2 melanoma cell lines, using the HLA-A2 negative M6 cell line as a control. In our melanoma collection, the M113 cell line displayed the highest level of recognition, presumably reflecting higher antigen expression (Figure 7A). However, even with this cell line, the level of recognition was variable possibly due to variable stress conditions in culture. We thus tested whether reticular stress induced by thapsigargin could enhance antigen expression on the M113 cell line, as we previously demonstrated for MELOE-1 expression.^34^ We observed that, despite persistent variability in response levels, thapsigargin-induced stress significantly enhanced the recognition of VS17p, SF15-dec1 and SF15-dec2 on M113 cells by their cognate T cells, supporting our hypothesis that translation and processing of these lincRNA-derived peptides is enhanced by stress in melanoma tumors *in vivo* (Figures 7B,C and S3).

### An ORF translation product from LINC00518 is present in melanoma cell lines

**Figure 8.**
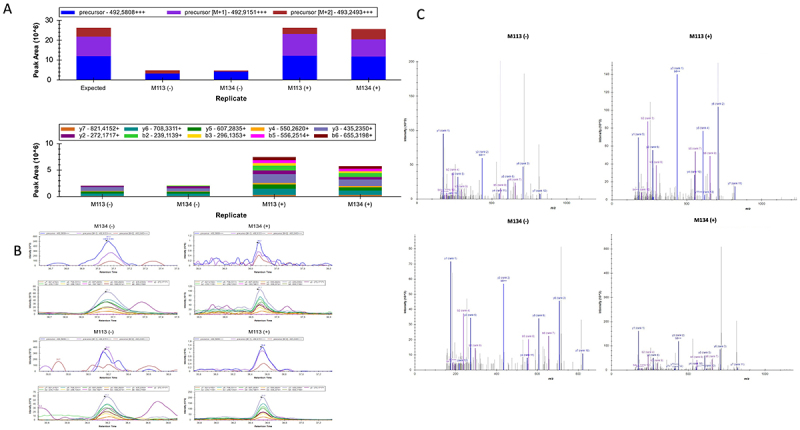
Detection of the translation of ORF549-905 from LINC00518 in melanoma cells by mass spectrometry. The peptide THGPYVITGDYPR (SRM5) was detected in total cell extracts from cells in thapsigargin treated (+) or untreated (-) conditions. (A) distribution of peak areas for precursor and transition ions of SRM5 across the different conditions. (B) identification spectra of precursor and transition ions based on retention time during the PRM analysis targeting SRM5. (C) Representative MS/MS spectrum of SRM5 showing the fragment ions used for identification, with labeled transitions corresponding to those reported in the supplementary table 2.

To ascertain that translation of ORF_549–905_ from LINC00518 occurred in melanoma cells, we performed mass spectrometry of melanoma cell lines M113 and M134, treated or not by thapsigargin. As shown in Figure 8 and Table S2, the precursor ions and ion transitions of the peptide “THGPYVITGDYPR,” specific for ORF549–905 from LINC00518, were detected. In unstimulated conditions [M] = 492.5808 and [M + 2] = 493.2493, were observed, whereas in the stimulated condition, a higher intensity was observed along with the presence of the precursor [M + 1] = 492.9151. Similarly, in both cell types, without thapsigargin stimulation, six transitions were detected, whereas ten transitions were observed after stimulation. Generally, three transitions per peptide are sufficient to confirm the presence of a peptide in PRM analysis. In the case of ORF_549–905_, we can therefore confirm its presence in both M113 and M134 cell types, before and after thapsigargin stimulation, with a notable increase in abundance under stimulated conditions. Altogether, we confirmed that translation of this ORF occurs in melanoma cells and was enhanced by stress.

## Discussion

In recent years, a growing body of evidence has demonstrated that transcript regions previously identified as “non-coding” contained small ORFs that could be translated into short peptides,^35–37^ some of which have physiological roles.^38^ Indeed, a significant number of lincRNAs are translated into peptides and should in fact be classified as coding RNAs.^39^ In parallel, a comprehensive analysis of the peptidome eluted from a human B lymphoblastoid cell line^40^ showed that up to 10% of the MHC-associated peptides originated from non-canonical translation of non-coding RNAs and that the majority of tumor-specific antigens originate from “non-coding” regions.^11^ However, despite accumulating evidence for the expression of such lincRNA-derived peptides in tumor cells,^5,14,16–18,35,41^ in most instances, the mechanisms regulating their translation and their immunological relevance as T cell epitopes in human cancers remain elusive.

Our previous findings on IRES-dependent translation of MELOE-1 from the lincRNA *meloe* prompted us to evaluate whether selecting ORFs from lincRNAs with IRES-like upstream structures could be a fruitful approach to systematically identify new tumor-specific antigens. As a proof of concept in melanoma, we selected transcripts identified as lincRNAs and expressed in both normal melanocytes and melanoma cell lines to set aside transcription regulations and focus on translation control. Our initial purpose in using SLP elongated from the predicted HLA-A *0201 epitope was to force the DC processing of the CD8 epitope. However, we noticed that our relatively short SLPs frequently triggered CD4 responses. A similar finding was reported by Ott *et al* in their vaccination trials with neoantigen SLPs designed around class I epitopes, but which triggered frequent CD4 responses in vaccinated patients.^6^ This indicates that class II-restricted epitopes frequently overlap class I-restricted epitopes as we previously documented.^42^

To favor cap-independent translation, we discarded the first long ORF of the transcript and searched for IRES-like secondary structures upstream of the subsequent ORFs using the mfold algorithm. Because there are few sequence homologies among the identified cellular IRES,^28^ we based our selection on the presence of stem-loops within the 200 nucleotides upstream that may accommodate IRES transactivating factors (ITAF), discarding very stable structures (γG < −85) in accordance with the comprehensive analysis of IRES reported by Weingarten-Gabbay *et al*.^29^

Using these selection criteria, 17/33 ORFs (51.5%) from 16/26 lincRNAs provided epitopes generating HLA-A *0201 restricted CD8 responses and/or CD4 responses in healthy donors. In our view, this high discovery rate validated our systematic strategy and suggested that translation of these ORFs did occur under certain conditions in healthy individuals. Moreover, since we restricted our search to HLA-A *0201 epitopes despite the presence of many potential epitopes for other frequent HLAs, the number of ORFs/lincRNAs producing T cell epitopes was probably greatly underestimated. We checked whether the recalled T cell responses toward SLPs may represent cross-reactive T cells but our thorough BLAST searches in the non-redundant protein sequence database on each SLP showed no significant homology with any other protein.

To assess the relevance of these antigens in melanoma, we examined the presence of specific T lymphocytes in TILs from patients with melanoma. We confirmed the presence of specific T lymphocytes against each identified epitopes in at least one TIL population. Moreover, we detected T cell repertoires against four selected epitopes in peripheral blood of patients with metastatic melanoma.

Finally, we provide evidence that T cell lines specific for these epitopes can recognize some of our melanoma cell lines *in vitro*. The low percentage of reactive T cells suggested that basal expression of the relevant HLA complexes was low on our melanoma cell lines. However, reticular stress, induced *in vitro* by thapsigargin, intended to mimic the stress conditions that tumor cells may encounter in *vivo* was able to greatly enhance T cell recognition, presumably through enhanced translation of the ORF. Accordingly, many previous reports have documented the links between stress and non-canonical translation in cancers.^43^ This hypothesis was confirmed by our MS analysis which confirmed the basal translation of the ORF_549–905_ of LINC00518 in the melanoma cell lines M113 and M134 which was significantly enhanced after thapsigargin treatment.

Concerning ORF_549–905_ of LINC00518, we showed that it provided epitopes that were recognized by TILs from 10/22 patients. In addition, a CD8 T cell repertoire against these peptides (especially SF15dec1 and dec2) was frequently found in 11/12 PBMC samples from melanoma patients. The corresponding LINC00518 is of particular interest: it is overexpressed in melanoma and has been suggested to promote metastasis by acting as a “miRNA sponge”^44,45^ and has recently been identified as a major survival factor for melanoma by promoting oxidative phosphorylation, and thus renamed Lenox.^46^ Finally, the peptide derived from the translation of ORF_549–905_ from LINC00518 was identified in two melanoma cell lines and its expression was enhanced by thapsigargin-induced reticular stress. Thus Lenox-derived peptides are particularly attractive candidates for melanoma immunotherapy.

Altogether, our data strongly support our identification strategy of lincRNA-derived T cell antigens in melanoma and we argue that this pipeline could be applied to other cancers, especially since some of the immunogenic lincRNA identified where also expressed in other tumor types. Whether these new antigens constitute promising new targets for immunotherapy of cancer need to be evaluated in *in vivo* models.

## Supplementary Material

SUPPLEMENTAL_FIGURE_LEGENDS.docx

## Data Availability

All data supporting our findings are available upon request. (francois.lang@univ-nantes.fr) Sequencing data are available at: https://data.mendeley.com/datasets/ntvc6bbffx/1.

## Material availability

Melanoma cell lines used in this report are available upon request (francois.lang@univ-nantes.fr).
