# Supplementary material for: Systematic identification of lincRNA-derived immunogenic peptides in melanoma
Source: Oncoimmunology. 2025 Aug 2;14(1):2538684. doi: 10.1080/2162402X.2025.2538684 (PMC12320848; doi:10.1080/2162402X.2025.2538684)
Supplement: SUPPLEMENTAL_FIGURE_LEGENDS.docx [file KONI_A_2538684_SM3768.docx]

SUPPLEMENTAL FIGURE LEGENDS

**Table 1 - Selected lincRNAs containing all the designed synthetic long peptides (SLPs).** SLP name, sequence, the ORF and the corresponding transcript are indicated. The last column shows sequences of the minimal immunogenic HLA-A*0201 identified epitopes. For each ORF, the mean of the 3 first ΔG values of the 200bp upstream of the ATG as predicted by mfold. All the immunogenic lincRNAs are shaded.

**Figure S1 -** Panel A: HLA-A2 restricted recognition of an SLP confirmed by inhibition of TNFa production with the addition of an HLA-A2 specific mAb (BB7.2).Panel B: Example of identification of the minimal HLA-A2 epitope within the selected immunogenic SLP. T cells reactive against SLP GV17 were re-challenged with T2 cells loaded with each potential HLA-A2 epitope and TNFa was assessed by intracellular staining. The decamer induced an optimal response as compared with the shorter corresponding nonamer-1 while nonamer 2 was not recognized.

**Figure S2**- Functional avidities of SF15-dec1, SF15-dec2 and VS17p specific T lymphocyte cell-lines were evaluated by measuring TNFα production in response to T2 cells loaded with a range of specific peptides. The EC50 (M) of peptide concentration for each tested T lymphocyte cell-lines are in brackets.

**Figure S3**- Recognition of melanoma cell-lines (pretreated or not with thapsigargin (200mM)) by T lymphocyte cell-lines specific for SF15dec1 and dec2. Representative experiments showing TNFa production (assessed by intracellular staining) of the T cell population after a 5h co-culture with the melanoma M6 (HLA-A2 negative) as control or M113 (HLA-A2 +). Gating was done on CD8+ cells to exclude melanoma cells from the analysis.

**Table S2 -** Detailed list of transitions observed for the PRM analysis of the peptide THGPYVITGDYPR (SRM5) in M113 and M134 melanoma cell-lines treated or not with thapsigargin. The table includes precursor ion information (charge state and m/z), associated fragment ions (b- and y-series), and their corresponding product ion m/z values. These transitions were used for the identification and validation of SRM5 and are aligned with the MS/MS spectrum shown in Figure 8C.
